# Coronary chronic total occlusion on coronary CT angiography: what radiologists should know?

**DOI:** 10.1186/s13244-024-01621-y

**Published:** 2024-02-27

**Authors:** Wei Xu, Junfeng Ma, Yiwen Chen, Fan Zhou, Changsheng Zhou, Long Jiang Zhang

**Affiliations:** 1 Department of Radiology, Jinling Hospital, Nanjing Medical University, 305 Zhongshan East Road, Nanjing, China; 2 Emergency Medical Center, Xi’an Xianyang International Airport Co., Ltd., Xianyang, China; 3 Department of Radiology, Affiliated Jinling Hospital of Medical School, Nanjing University, 305 Zhongshan East Road, Nanjing, China

**Keywords:** Chronic total occlusion, Coronary computed tomography angiography, Percutaneous coronary intervention

## Abstract

Coronary chronic total occlusion (CTO) often occurs in patients with obstructive coronary artery disease, which remains one of the greatest challenges for interventional cardiologists. Coronary computed tomography angiography (CCTA) with its emerging post-processing techniques can provide a detailed assessment of CTO lesions before percutaneous coronary intervention (PCI), playing an important role in the clinical management of CTO PCI, from early diagnosis, pre-procedural outcome prediction, the crossing algorithm planning, intraprocedural guidance, and finally post-procedural assessment and follow-up. In addition, the feasibility of CT perfusion (CTP) in patients with CTO has been validated. Combined CCTA and CTP have the great potential to be the one-stop-shop imaging modality for assessing both anatomy and function of CTO lesions. This review aims to make radiologists understand the role of CCTA in the diagnosis and assessment of CTO lesions, thus assisting interventionalists in optimizing CTO PCI crossing strategies with the expertise of radiologists.

**Critical relevance statement** The anatomical features of CTO on CCTA can reveal the complexity of CTO lesions and are associated with CTO PCI outcome, thus helping interventionalists optimize CTO PCI crossing strategies.

**Key points** • CTO is the common lesion in invasive coronary angiography, and CTO PCI is technically difficult and its success rate is relatively low.

• Length, collaterals, and attenuation-related signs can help distinguish CTO from subtotal occlusion.

• The anatomical features of CTO lesions can help grade the difficulty of CTO PCI and predict procedural outcomes and long-term outcomes of CTO PCI.

• The real-time fusion of CCTA with fluoroscopic angiography can be applied in highly complicated CTO lesions.

• After CTO PCI, CCTA can help guide a second CTO PCI re-entry or follow up stent patency.

**Graphical Abstract:**

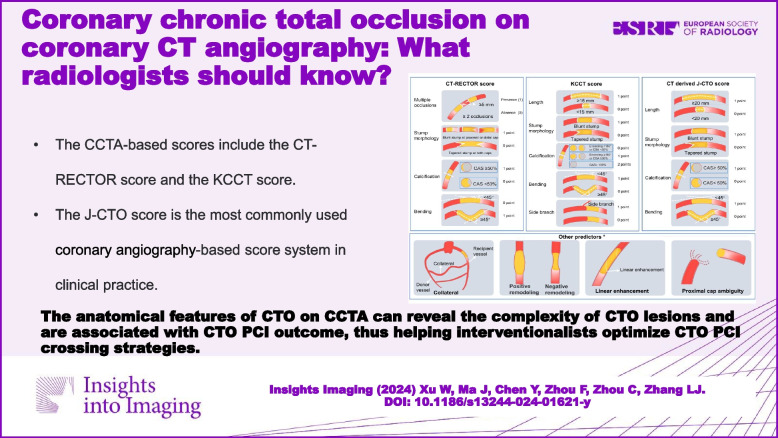

## Introduction

Coronary chronic total occlusion (CTO) is defined as a total occlusion of coronary artery on invasive coronary angiography (ICA) with thrombolysis in myocardial infarction (TIMI) of 0 flow for ≥ 3 months [1]. It has been confirmed that 15 to 26% of patients referred for ICA had CTO [2–4]. In previous clinical practice, coronary artery bypass grafting and optimal medical therapy have been the main treatment strategies for CTO lesions [2]. With the rapid advancement of percutaneous coronary intervention (PCI) techniques, PCI has been increasingly used to treat CTO lesions in recent years. However, CTO PCI remains one of the most challenging procedures for interventional cardiologists, often described as “the last frontier,” mainly due to low procedural success rates, high incidence of procedural complications, and controversial clinical benefits [1, 5, 6]. Intraprocedural evaluation by ICA often has problems such as incomplete assessment of CTO lesions, long procedural time, and excessive use of contrast agents. In recent years, advanced modern imaging modalities, especially coronary computed tomography angiography (CCTA), have been used to manage CTO PCI from pre-procedural assessment to post-procedural guidance, thus reducing procedural time, optimizing the strategy of CTO PCI and improving procedural success rate. Thus, it is necessary to introduce basic knowledge of CTO on CCTA to radiologists, thus efficiently guiding clinical practice.

## Methods

A comprehensive PUBMED search was performed. The search date ends in September 2023. The following terms, including (chronic total occlusion OR CTO) AND (coronary occlusion OR coronary stenosis OR coronary disease) AND (percutaneous coronary intervention OR PCI), were searched and 3258 results were obtained. A further search was performed with the following terms: (chronic total occlusion OR CTO) AND (coronary occlusion OR coronary stenosis OR coronary disease) AND (percutaneous coronary intervention OR PCI) AND (computed tomography angiography); (chronic total occlusion OR CTO) AND (coronary occlusion OR coronary stenosis OR coronary disease) AND (pathology OR histology); (chronic total occlusion OR CTO) AND (coronary occlusion OR coronary stenosis OR coronary disease) AND (percutaneous coronary intervention OR PCI) AND (score system OR predictor OR predicting); (chronic total occlusion OR CTO) AND (coronary occlusion OR coronary stenosis OR coronary disease) AND (percutaneous coronary intervention OR PCI) AND (crossing algorithm); and 150, 424, 800, and 35 results were obtained, respectively. After removing repetition, we reviewed the basic knowledge of coronary CTO that radiologists need to know according to the literature and our experiences.

## Results

### Clinical and pathological features of CTO lesions

Some CTO patients have mild symptoms or are asymptomatic, which may be related to the formation of collateral circulation and neovascularization that can maintain part of the myocardial perfusion [2, 7]. However, although some CTO patients with well-developed collateral circulation, the symptoms of myocardial ischemia could still occur with the increase of myocardial burden [8]. CTO lesions commonly occur in the right coronary artery and are often accompanied by multi-vessel diseases [2].

CTO lesions could originally be derived from acute occlusion due to rupture of high-risk plaque, or from the progression of atherosclerosis [7, 9]. CCTA features can help identify risk factors for CTO progression. Kang et al. compared CCTA baseline features between the future CTO group and non-CTO group and showed that patients with small minimal lumen diameter, small reference segment diameter, and low mean plaque attenuation were more likely to progress into CTO lesions [10].

**Fig. 1:**
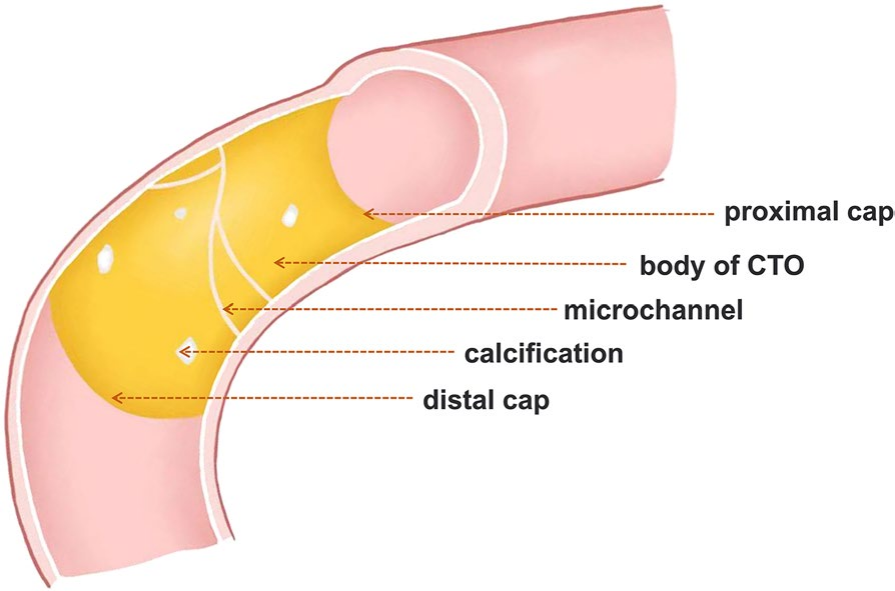
The components of chronic total occlusion (CTO) lesions

With the age of CTO, thrombosis or plaque gradually fiberizes and organizes, progressing into fibrosis, calcification, and neovascularization or microchannels [7]. Additionally, with blood flow shocking, dense fibrous caps are formed at the proximal and distal lesions. Thus, CTO lesions usually consist of 3 components [11]: the proximal cap, the body of CTO, and the distal cap (Fig. 1). The composition of the body of a CTO can be classified as “hard,” “soft,” or “mixed,” accounting for 64%, 11%, and 25%, respectively, which can change with the increase of CTO age [7, 9]. CCTA can reveal such time-dependent progression of CTO lesions by displaying plaque attenuation [12, 13].

### CCTA for diagnosing CTO lesions

CTO is featured as a complete absence of contrast opacification within the occluded segment on CCTA [14]. However, it is challenging for radiologists to distinguish CTO from subtotal occlusion (STO) on CCTA. Two main reasons could explain this challenge. Firstly, severe calcification or limited spatial resolution of CT may result in mistaking STO for CTO [14–16]. Secondly, linear enhancement in CTO also makes radiologists confuse CTO with STO. Histologically, CTO is not always what is called “total occlusion” due to the presence of microchannels or recanalized lumen. A previous study showed that only 22% of ICA-confirmed CTOs were totally occluded [7]. This microvascular ranges from 100 to 500 μm and could partially appear as a linear enhancement on CCTA [12, 17]. Therefore, radiologists should pay attention to the presence of linear enhancement of CTO. The differentiation between CTO and STO is of clinical value as CTO often indicates a poorer prognosis and more technical difficulties in PCI than STO [15]. Some CT-derived signs can help us identify CTO lesions comprehensively; see the following subsections.

#### Length

CTO length is usually longer than STO. Four CCTA studies provided 4 different thresholds (i.e., 1 mm, 9 mm, 14.3 mm, 15.0 mm) [14, 15, 18, 19]. In the study of von Erffa et al. [19], length (cutoff value: 9 mm) was the only factor to distinguish CTO from STO. Subsequently, Li et al. and Choi et al. [14, 15] concluded 14.3 mm (area under the curve (AUC) = 0.912) and 15 mm (c-statistics, 0.732) as the best cutoff values, respectively. In their studies, length had a higher predictive performance than other parameters.

#### Collaterals

CTO lesions usually have more visualization of collaterals than STO. However, the incidence of collaterals in CTO lesions is not high on CCTA. In Li et al.’s and Choi et al.’s studies [14, 15], collaterals were present in 8% (4/49) and 39.4% (162/411) of cases, respectively. With the improved CT spatial resolution in recent years, collaterals have been increasingly displayed on CT, helping precisely distinguish CTO from STO.

#### Attenuation-related signs

Normally, the attenuation of coronary artery from the proximal to the distal segment is constant or only slightly or gradually decreases [20]. However, for CTO artery, the attenuation of the distal vessel is often higher than that of the vessel near the occlusion site. Transluminal attenuation gradient (TAG) can be used to reflect the change in attenuation with distance on CCTA and was determined from the change in Hounsfield units per 10-mm length of coronary artery. The cutoff of TAG = 0 HU/10 mm can discriminate the antegrade and retrograde flow in distal vessels (c-statistics, 0.88) [20, 21]. Choi et al. [14] used CCTA to measure the anatomical features and TAG in the large clinical population and showed TAG ≥  - 0.9 HU/10 mm was the optimal threshold for distinguishing CTO from STO (c-statistics, 0.678). Based on TAG analysis, Li et al. [15] proposed the reverse attenuation gradient (RAG) that was defined as a reverse luminal opacification gradient of distal vessels to occlusion site (TAG > 0 HU/10 mm). They found that CTO had more RAG signs than STO (65% vs. 7%, *p* < 0.01), and RAG is a highly specific CTO sign (specificity, 93%), representing reverse filling of the reverse collaterals. However, 35% of CTOs still lacked RAG sign. Two reasons can explain this phenomenon, one is that bridging collaterals cause antegrade filling to the distal segment of CTO; the other is that the late phase of the scan may lead to all segments distal to the occlusion being filled by retrograde collaterals.

#### Comprehensive assessment

**Fig. 2:**
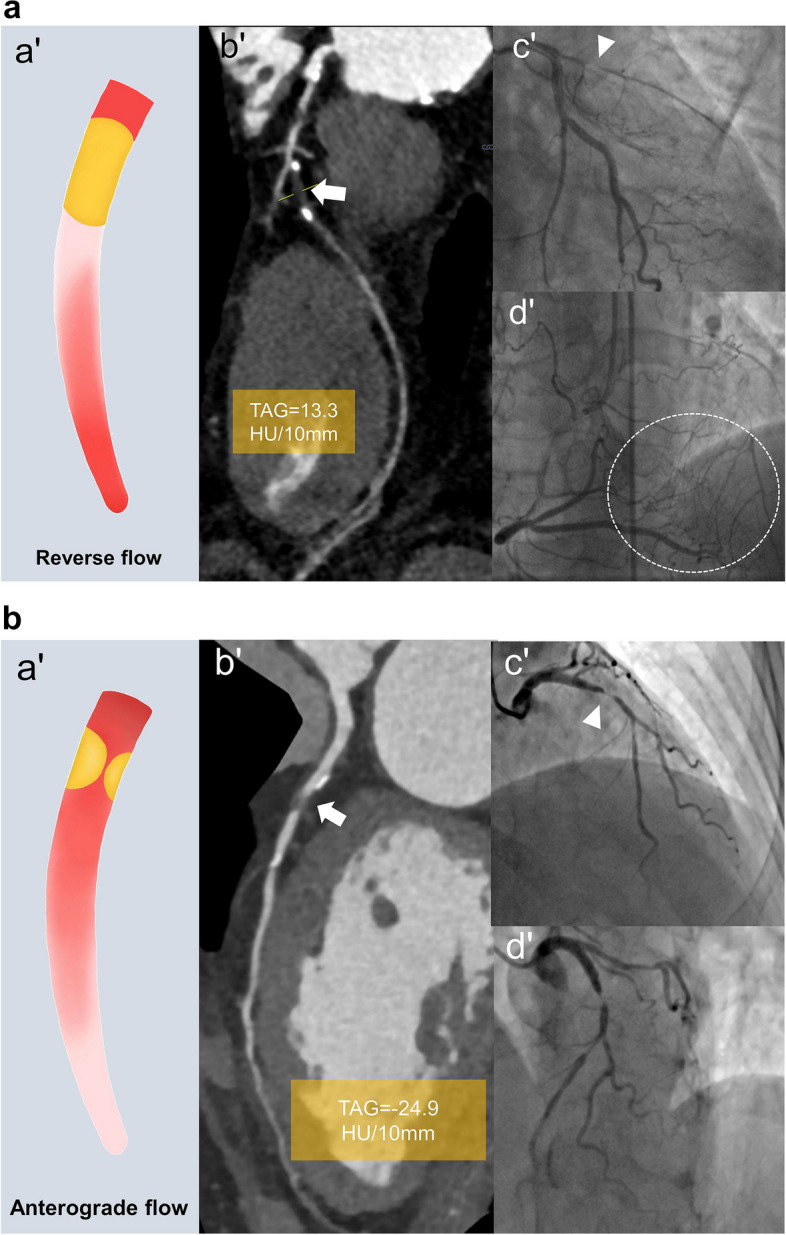
Representative images of CTO and subtotal occlusion (STO). **a** CTO: (a′) Illustration of the reverse flow of CTO. (b′, c′) Curved planar reformation image demonstrates a 16.4-mm non-opacified CTO lesion in the proximal left anterior descending artery (LAD) (arrow), shown in invasive coronary angiography (ICA) (arrowhead). (d′) The mid and distal LAD is displayed via collateral channel from the right coronary artery (RCA) on ICA (circle). Transluminal attenuation gradient of distal segments = 13.31 HU/10 mm, indicating the presence of the reverse attenuation gradient (RAG) sign. **b** STO: (a′) Illustration of the anterograde flow of STO. (b′, c′) Curved planar reformation image demonstrates a 4.9-mm non-opacified STO lesion in the proximal LAD (arrow), shown in ICA (arrowhead). (d′) There are no visible collateral channel. Transluminal attenuation gradient of distal segments =  - 24.94 HU/10 mm, indicating the absence of RAG sign

Comprehensive analysis of the abovementioned CCTA signs can further improve the diagnostic accuracy, the combination of the above three signs could output a higher diagnostic accuracy (92%) in distinguishing CTO from STO [15] (Fig. 2). In Choi et al.’s study [14], the combination of TAG, some anatomic features, and collaterals yielded a higher performance in predicting CTO (c-statistics = 0.88). New markers or automated tools integrating multi-scale information are urgently needed to accurately identify CTO in the future.

### The indication of CTO PCI

Two groups of patients are recommended for CTO PCI in the guidelines. Firstly, for patients with angina resistant to medical therapy, improvement of symptoms is the primary indication for CTO PCI, which is now well-established by randomized trials and recommended in current guidelines [22–25]. Secondly, for patients without symptoms, the 2018 ESC/EACTS guideline recommended CTO PCI in patients with a large area of ischemia in the territory of viable myocardium [25]. Several studies using non-invasive cardiac functional imaging tools (SPECT, PET, MR) [26–28] showed that patients with evidence of myocardial ischemia and viability may have potential benefits after CTO PCI. A previous study showed that baseline ischemia of ≥ 12.5% was associated with a significant decrease in ischemic burden after CTO-PCI [26]. However, using myocardial functional parameters as the indication to perform CTO PCI remains debated due to the limited evidence of improving hard outcomes [3, 29] and the lack of cut-off values of ischemia and viability quantification in patients with CTO. The CARISMA_CTO study [30] and the ongoing ISCHEMIA-CTO trial [31] can provide more definitive answers in this area shortly.

Routinely, it is recommended that non-invasive CT-fractional flow reserve (FFR) can detect functional implications of 30−90% diameter stenosis in clinical practice [32, 33]. However, CT-FFR cannot be used in CTO lesions [25]. CT perfusion (CTP) can assess myocardial ischemia in patients with coronary artery disease or look for viable myocardium around infarcted myocardium to determine whether patients have indications for PCI. Kwiecinski et al. [34] first introduced dynamic CTP into CTO patients; such dynamic CTP together with CCTA can obtain anatomical and perfusion data in a single imaging without two independent scans. The study confirms that dynamic CTP is a robust and safe approach that provides a truly practical one-stop workflow for the anatomical and functional evaluation of CTO PCI. However, larger samples and multimodal imaging are still needed for further validation.

### CCTA for preprocedural prediction of CTO PCI procedural outcomes

#### A general introduction to specific CT findings linked with procedural outcomes

CCTA can evaluate the CTO morphology that could reveal the CTO complexity to help interventional cardiologists choose the appropriate surgical strategies or guidewires [35]. Several previous studies have shown that CCTA before CTO PCI can improve the success rate of CTO PCI. The first randomized controlled study randomized 400 patients to the CCTA-guided group and angiography-only group and found that the success rate of the CCTA-guided group was significantly higher than that of the angiography-only group (93.5% vs. 84.0%) [35]. However, the study included patients with all Japanese-chronic total occlusion (J-CTO) scores [35, 36]; an upcoming randomized controlled study (NCT04549896) [37] will further explore the effect of preprocedural CCTA for planning CTO PCI for complex CTO (J-CTO score > 2) lesions.

#### Predictors of CTO PCI procedural outcomes

##### Calcification

Calcification poses difficulties at all CTO PCI steps since it may impede proper guidewire passage, pre-dilatation, and stent expansion [38–40]. CCTA is more sensitive than ICA for detecting, measuring, and localizing calcification [40, 41], thus helping predict CTO PCI procedural success. Firstly, calcification area covering > 50% of the vessel cross-section area measured on CCTA was commonly used to be the severe calcification benchmark to predict CTO PCI success [38, 40, 42, 43]. Secondly, due to the difficulty of quantifying the calcification area, the calcification/lesion length ratio > 0.5 was used to predict CTO PCI success [39]. In addition, some studies based on circular involvement of calcification to assess the degree of calcification of CTO lesions [35], such as calcification arc on cross-section ≤ 180°, > 180°, 360°, or none. Besides the degree of calcification, the distribution of calcification also impacts the outcome of PCI [44], since the central calcification may make it more difficult to advance the wire than circular wall calcification with a central softcore.

##### Stump morphology

**Fig. 3:**
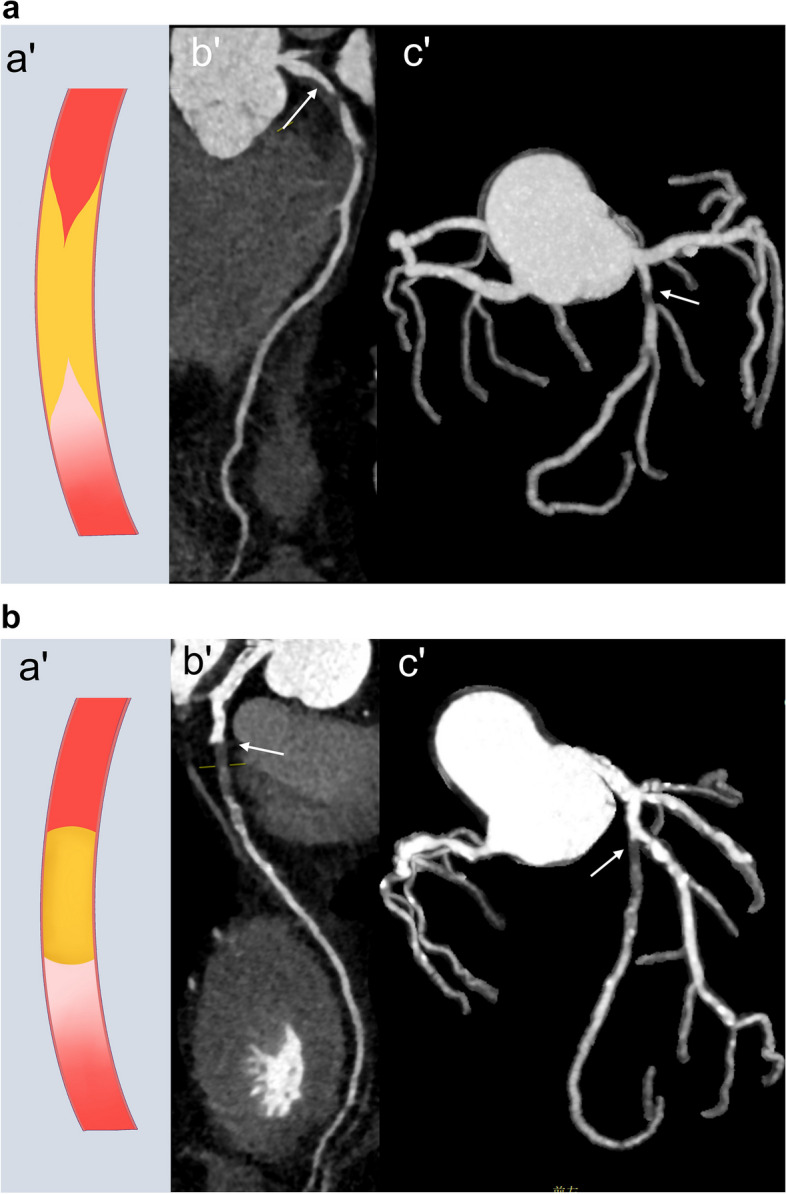
Examples of tapered and blunt stump in successful and failed CTO percutaneous coronary intervention (PCI). **a** Tapered stump: A 38-year-old man with chest pain for more than 6 months, (a′) Illustration of tapered stump. (b′, c′) Curved planar reformation and maximum intensity projection images demonstrate a non-opacified CTO lesion with a tapered stump in the proximal cap of CTO (arrow). The patient has a successful PCI. **b** Blunt stump: A 39-year-old man with chest pain for more than 1 year, (a′) Illustration of blunt stump. (b′, c′) Curved planar reformation and maximum intensity projection images demonstrate a non-opacified CTO lesion with a blunt stump in the proximal LAD (arrow). After several attempts, the guidewire did not cross the LAD occluded lesion

The morphology of the proximal or distal caps of CTO lesion was classified as a tapered stump if the occlusion terminated in a funnel-shaped form, or it could be defined as a blunt stump on the maximum intensity projection image of CCTA [43]. The tapered stump usually has looser fibrous tissue with obvious neovascularization and recanalization compared with blunt stump, making it easier for a guidewire to enter the actual distal lumen inside microchannels [9, 11]. Multiple studies [36, 42, 44, 45] showed that the stump appearance, especially the stump of the proximal cap, was significantly associated with procedural success by multivariable regression analyses of CTO variables (Fig. 3). Moreover, several studies also revealed that CCTA was more sensitive than ICA to identify blunt stump because the time required for CCTA could permit the contrast agent to remain in the microchannel within the CTO lesions [41, 43].

##### Length and multiple occlusions

The longer the CTO lesion, the more difficult it is for the guidewire to pass through the lesion. Continuous loose tissue is often present in the short CTO [9, 11], which may facilitate the guidewires crossing. Occlusion length ≥ 20 mm based on ICA has been widely used as the cutoff value for predicting the outcome of CTO PCI, which is routinely recommended by the Euro CTO Club [36, 45–47]. Length ≥ 15 mm or 32 mm measured by CCTA was also found to predict the difficulty of guidewire crossing [44, 48]. Such different cutoff values may be attributed to the different competencies of operators in handling long CTO lesions. In addition, Opolski et al. [42] found that multiple occlusions which are defined as more than 2 total occlusions separated by contrast-enhanced segments of ≥ 5 mm on CCTA could potentially exceed the discriminatory accuracy of length for predicting guidewire crossing.

##### Bending

Bending may increase the difficulty in passing guidewire through the occluded lesion and lead to more severe coronary artery injury. CCTA can depict the vessel contour and course by the maximum intensity projection, enabling precise measurement of tortuosity of the occluded coronary artery. It was often defined as at least one bend > 45° within the CTO lesions and was the predictor of CTO PCI failure widely recognized by most scholars [36, 42–44].

##### Remodeling

**Fig. 4:**
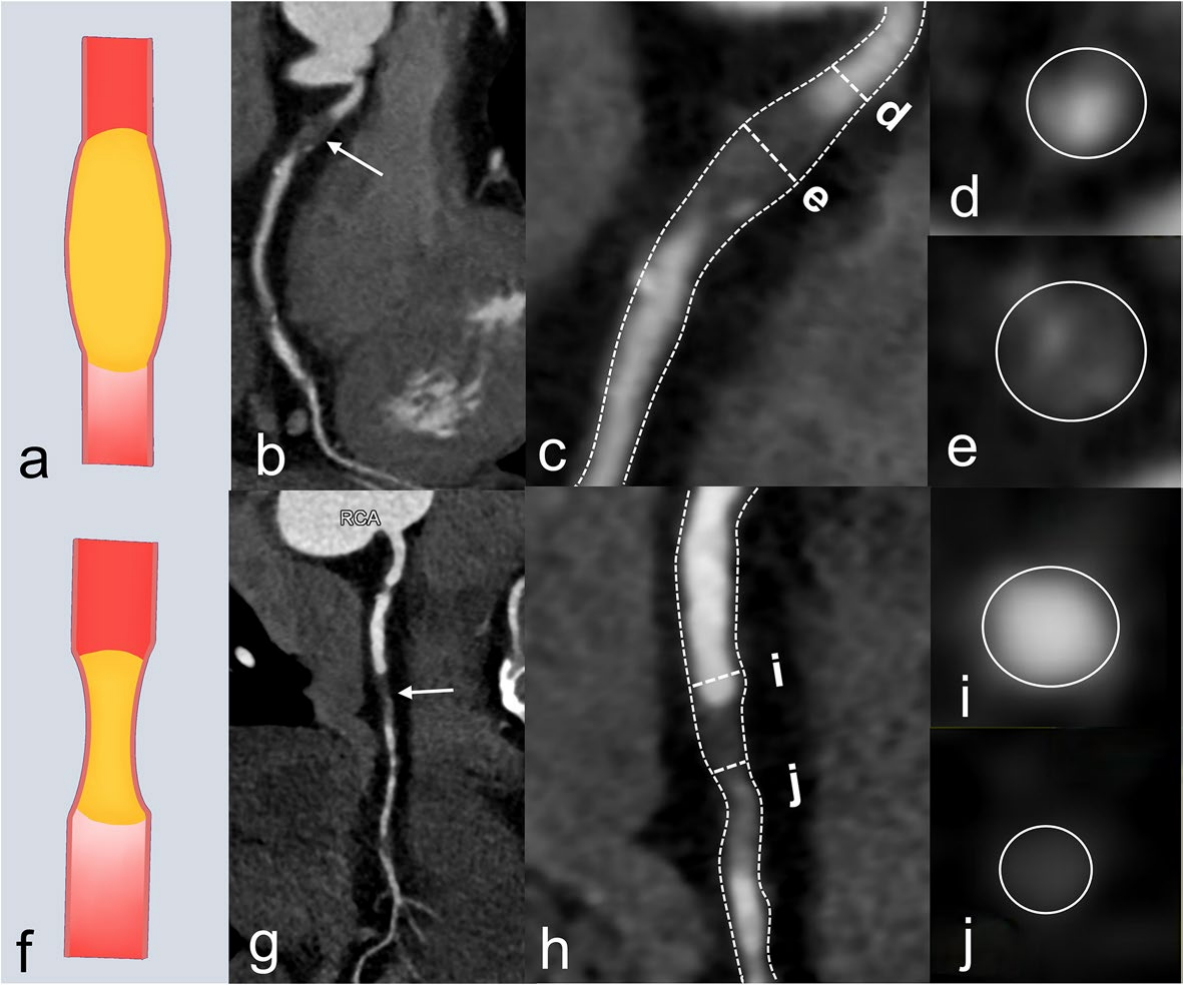
Representative examples of CTO remodeling. **a** Illustration of positive remodeling. **b** Curved planar reformation image showing CTO (arrow) of the proximal LAD. **c** Maximal CTO segment vessel diameter was larger than the reference diameter. **d** Axial CCTA image of proximal reference. **e** Axial CCTA image showing maximum vessel diameter in the CTO segment. **f** Illustration of negative remodeling. **g** Curved planar reformation image shows CTO (arrow) of the middle RCA. **h** Maximal CTO segment vessel diameter was less than the reference diameter. **i** Axial CCTA image of proximal reference. **j** Axial CCTA image showing maximal vessel diameter in the CTO segment

CTO segment remodeling can be assessed by multi-planar reformation compared with proximal reference segments on CCTA, which could help predict CTO PCI success (Fig. 4). Luo et al. [48] found that negative remodeling on CCTA was the strongest predictor of failed antegrade-PCI (odds ratio = 137.82). Moreover, Yamamoto et al. [49] classified the CTO segment remodeling into 3 categories based on CCTA: positive remodeling, non-positive remodeling, and collapse. Such classification may affect the choice of surgical strategy. They found that the collapsed group required more retrograde approach with more radiation and time than the non-collapsed group.

##### Linear enhancement

**Fig. 5:**
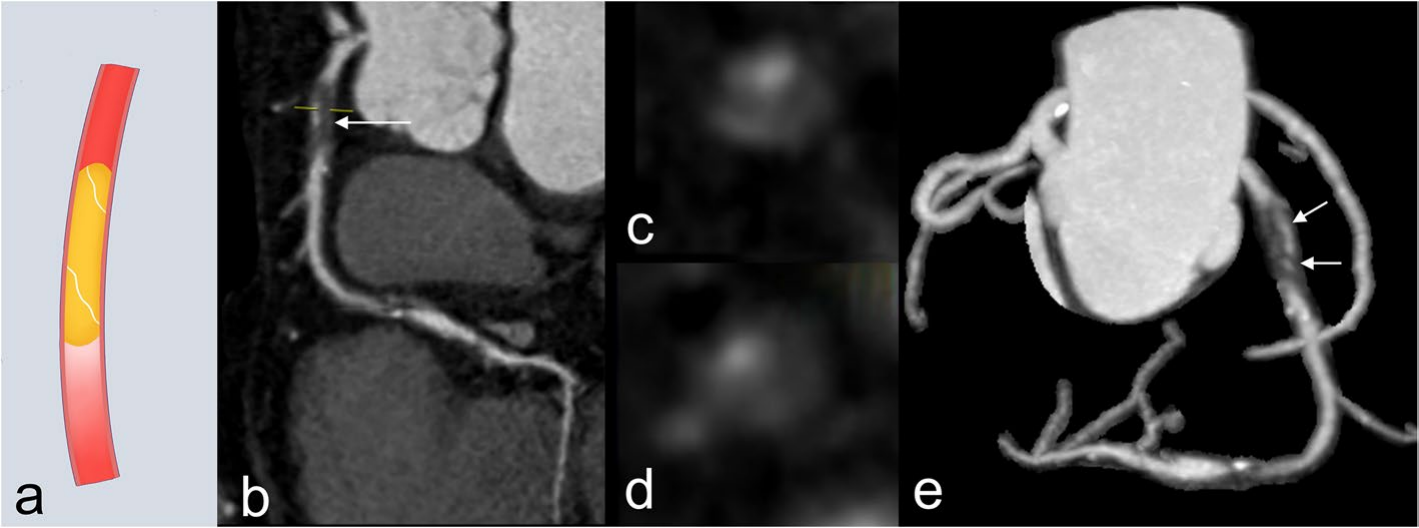
CTO lesion with linear enhancement on CCTA. **a** Illustration of linear enhancement. **b** Curved planar reformation image shows CTO (arrow) of the proximal LAD. **c**, **d** Axial CCTA images of CTO segment show the presence of the tiny linear enhancement. **e** Maximum intensity projection image shows the proximal LAD CTO lesion with discrete linear enhancement (arrows)

The microvascular, partially appearing as a linear enhancement on CCTA, is the hallmark feature of CTO (Fig. 5). Microvasculature not only provides blood flow to supply the myocardium subtended by CTO but also could predict CTO PCI outcomes [17, 50]. In Li et al.’s [17] study, linear enhancement of CTO was present in 59% of successful CTO PCI, and the number was only 19% in failed CTO PCI. Furthermore, the plaque around the microvascular is loose, which is conducive to the passage of guidewires [9, 50]. Recently, Lee et al. [51] utilized spectral CT to evaluate the coronary iodine concentration at the proximal segment of CTO and found that low coronary iodine concentration (< 2.5 mg/mL) is independently associated with unsuccessful antegrade PCI. It could be hypothesized that the iodine concentration may be correlated with the formation of microvascular and softer plaque. More experimental or clinical studies with larger samples are required to identify the exact nature of iodine concentration and its relationship with microvascular at the proximal segment of CTO.

##### Proximal or distal side branch

Proximal or distal side branch is defined as the presence of any side branch within 3 mm proximal to the entry or exit of the occlusion. CCTA can display the anatomical relationship between the side branch and CTO lesions by various projecting angles. The side branch is a frequent predictor of CTO PCI failure [52], as the guidewire may cross the side branch instead of the CTO lesions [42].

##### Collateral channel

**Fig. 6:**
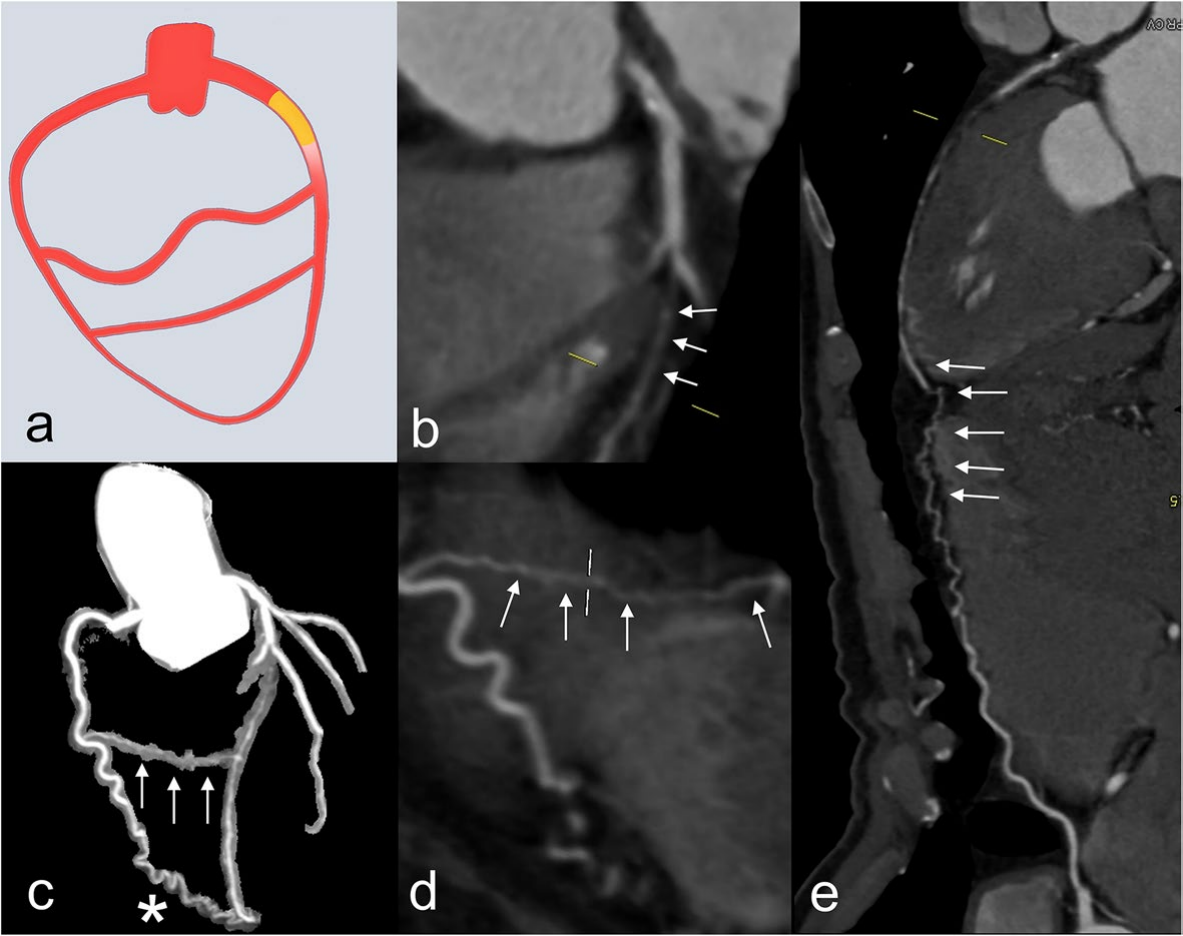
Representative images of CTO with septal and epicardial collateral channel. **a** Illustration of collateral channels. **b** Curved planar reformation image shows CTO (arrows) of the middle LAD. **c** Maximum intensity projection image shows the septal (arrows) and the epicardial (asterisk) collateral channel of CTO lesion from RCA to LAD. **d** A continuous connection of CTO septal collateral channel (arrows) from RCA to LAD is seen on curved planar reformation image. **e** A continuous epicardial collateral (arrows) from RCA to LAD is seen on the curved planar reformation image

The anatomical morphology of collateral channel (Fig. 6) (the complete connection between donor and recipient arteries) is also an essential issue for a successful retrograde approach [53]. The collateral channels are usually classified into 3 types: septal, epicardial, and atrioventricular groove. Traditionally, the Werner classification of collateral connection [54] and the Rentrop grade [55] are used to assess the diameter and filling of collateral channels. In CTO PCI, the elements of collateral channel evaluation include the length, bending, side branch, and distance between the collateral exit and the CTO distal cap [56, 57]. The J-Channel score based on the size and bending of the collateral channel measured on ICA was created to predict the difficulty of guidewires crossing and the retrograde CTO PCI success [58]. It is challenging for CCTA to provide detailed information regarding collateral channels in retrograde PCI due to its limited spatial resolution [59]. In the study of Sugaya et al. [60], the accuracy of CCTA for detecting collaterals used for the retrograde PCI was 74.5%, and a higher success rate with fewer complications was found in CCTA-visible collaterals than in those not apparent in CCTA. Nowadays, photon-counting CT with a spatial resolution of around 0.25 mm [61] may be potentially precise to assess collateral channels.

##### Proximal cap ambiguity

Proximal cap ambiguity is defined as an inability to determine the precise location of the proximal cap of CTO lesions, which is present in almost one-third of CTOs and was associated with the failed CTO PCI [62–64]. It is a crucial parameter utilized to decide whether the dominant approach is antegrade or retrograde according to current CTO crossing algorithms [46, 65–68]. CCTA can be potentially applied for clear visualization of the proximal cap that the ICA findings are inconclusive [56]. For example, Simsek et al.’s study showed that CCTA can help clearly show 27% of proximal cap ambiguity on ICA [69].

**Fig. 7:**
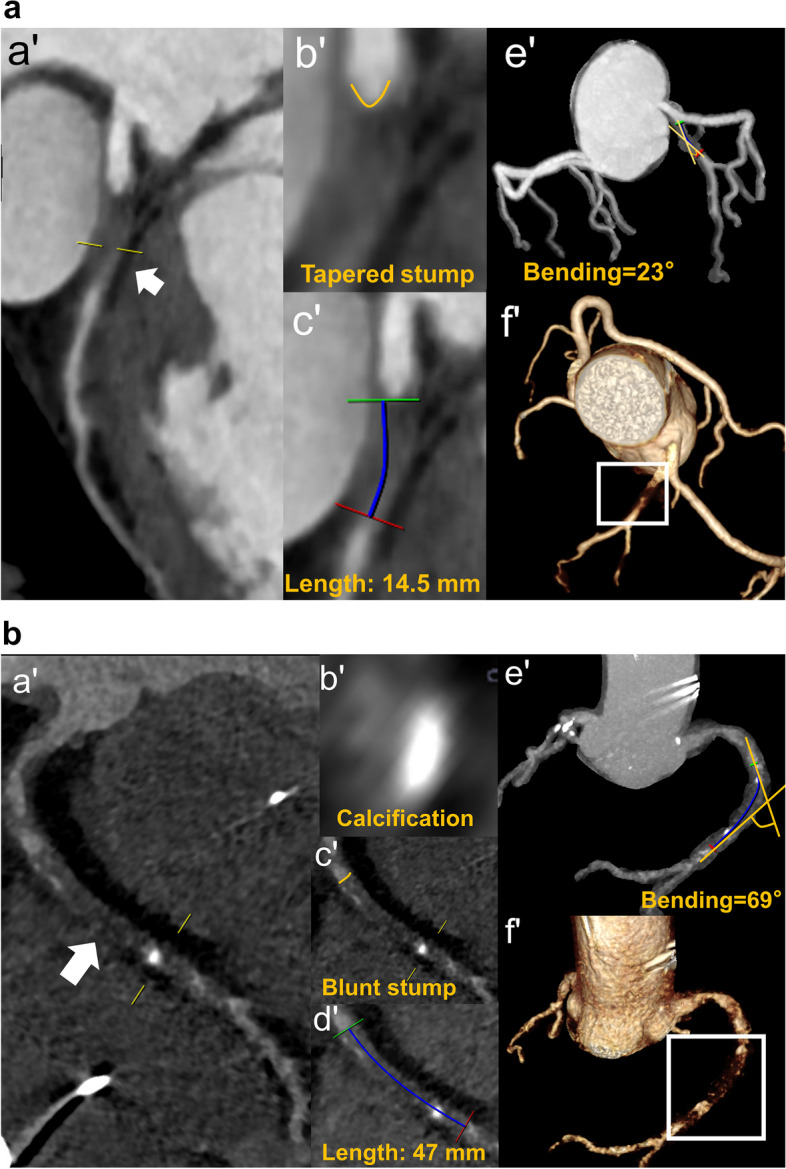
Examples of CTO lesions in successful and failed CTO PCI. **a** Successful CTO PCI: A 61-year-old man with chest pain for more than 4 months. (a′, c′, f′) Curved planar reformation image demonstrates a 14.5-mm non-opacified CTO lesion in the proximal LAD (arrow), also shown in volume rendering (quadrilateral). (b′, e′) The CTO lesion had a tapered stump and bending < 45°. **b** Failed CTO PCI: A 79-year-old man with chest pain for more than 1 year. (a′, d′,f′) Curved planar reformation image demonstrates a 47-mm non-opacified CTO lesion in the middle RCA (arrow), shown in volume rendering image (quadrilateral). (b′, c′, e′) The CTO lesion had a blunt stump, bending > 45°, and the cross-section area of calcification < 50%

In summary, predictors measured by CCTA have a promising potential in predicting successful CTO PCI or guidewire crossing (Fig. 7), especially for lesions that require particularly complicated CTO PCI techniques. Of note, the above morphological parameters were not always predictors of CTO PCI outcomes in each study, due to heterogeneity in the clinical background, such as different PCI strategies, techniques, equipment, and operators’ experience.

#### CTO scoring systems

**Fig. 8:**
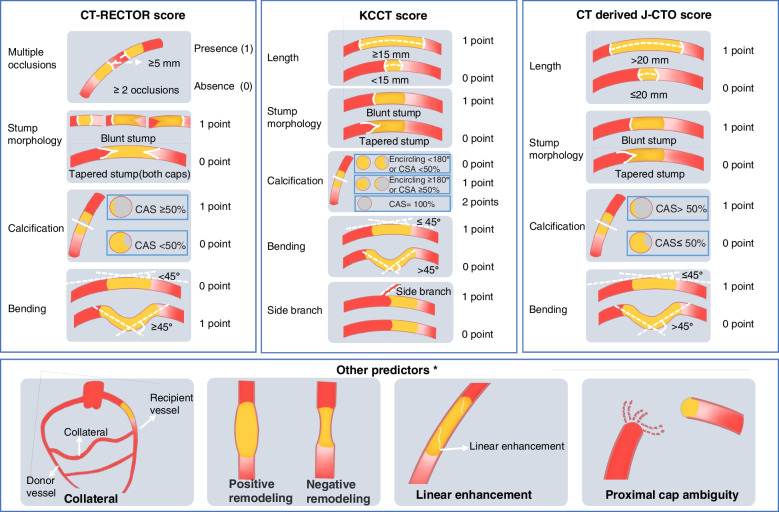
Scoring systems on CCTA and other predictors. CT-RECTOR, Computed Tomography Registry of Chronic Total Occlusion Revascularization; KCCT, Korean Multicenter CTO CT Registry; J-CTO, Multicenter CTO Registry of Japan; CAS, cross-sectional area. *Predictors not mentioned in the score systems based on CT

Different score systems (Table 1) have been developed to grade the difficulty of CTO PCI. The J-CTO score [36] is the first system used to predict the difficulty of passing a guidewire and is now widely recognized and used in clinical practice, which includes 4 variables: calcification, stump morphology, length, and bending. Subsequently, some other scores based on ICA [45, 47, 58, 70–74] were created to apply for different PCI crossing strategies. CCTA can precisely provide detailed anatomical characteristics of CTO lesions and thus propose several score systems based on CCTA (Fig. 8), such as the CT-RECTOR score (Computed Tomography Registry of Chronic Total Occlusion Revascularization) [42], the KCCT score (Korean Multicenter CTO CT Registry) [44], and other CT-derived scores.

**Table 1:** Research summary of CTO scoring systems

	**Scores**	**Year**	**Sample**	**Primary endpoint**	**Performance**	**Clinical variables**	**Morphological variables**	**Application**
Scores based on CCTA	CT-RECTOR [42]	2015	229	Guidewire crossing within 30 min	AUC = 0.83	Previous attempt but failed (+ 1), duration of CTO ≥ 12 months or unknown (+ 1)	Calcification (+ 1), blunt stump (+ 1), bending ≥ 45° (+ 1), multiple occlusion (+ 1)	Antegrade
	KCCT [44]	2017	643	Guidewire crossing within 30 min	c-statistics = 0.78	Previous attempt but failed (+ 1), duration of CTO ≥ 12 months or unknown (+ 1)	Calcification (+ 1/2), blunt stump (+ 1), bending > 45° (+ 1), proximal branch (+ 1), occlusion length ≥ 15 mm (+ 1)	Antegrade
	CTAP [52]	2022	201	Guidewire crossing within 30 min	c-statistic = 0.81	Previous attempt but failed (+ 1)	Blunt stump (+ 1), bending > 45° (+ 1), proximal branch (+ 1), distal branch (+ 1), high-density plaque volume ≥ 19.9 mm^3^ (+ 1)	Antegrade
Radiomics model [75]		2023	375	Technical success	AUC = 0.920		CT radiomics model	Uncertain
Scores based on ICA	J-CTO [36]	2011	465	Guidewire crossing within 30 min	AUC = 0.82	Previous attempt but failed (+ 1)	Calcification (+ 1), blunt stump (+ 1), bending > 45° (+ 1), occlusion length ≥ 20 mm (+ 1)	Antegrade
	CL [47]	2015	1657	Technical success	AUC = 0.68	Previous CABG (+ 1.5), previous myocardial infarction (+ 1)	Calcification (+ 2), blunt stump (+ 1), occlusion length ≥ 20 mm (+ 1.5), non-LAD CTO location (+ 1)	Antegrade
	ORA [71]	2016	1019	Technical success	AUC = 0.728	≥ 75 years (+ 1)	Collateral filling (Rentrop 0–1) (+ 2), ostial location (+ 1)	Hybrid
	PROGRESS-CTO [70]	2016	781	Technical success	AUC = 0.778		2 bends > 70°or 1 bend > 90 (+ 1), no interventional collaterals (+ 1), proximal cap ambiguity (+ 1), circumflex artery CTO (+ 1)	Hybrid
	RECHARGE [45]	2018	880	Technical success	AUC = 0.783	Previous CABG on target vessel (+ 1)	Calcification (+ 1), blunt stump (+ 1), bending > 45° (+ 1), occlusion length ≥ 20 mm (+ 1), diseased distal landing zone (+ 1)	Hybrid
	CASTLE [72]	2019	> 20,000	Technical success	AUC = 0.66	Previous CABG (+ 1), age ≥ 70 (+ 1)	Calcification (+ 1), blunt or invisible stump (+ 1), severe bending or unseen (+ 1), occlusion length ≥ 20 mm (+ 1)	Hybrid
	Clinical Prediction [73]	2016	223	Technical success	AUC = 0.832		Werner’s score < 1.5 (+ 1), diameter of distal CTO segment < 1.48 (+ 1), tortuous collateral > 0.5 (+ 1)	Retrograde
	J-Channel [58]	2020	630	Technical success	AUC > 0.74		CC vessel size (+ 2/3), reverse bend (+ 1), continuous bends (+ 1/0), corkscrew (+ 1/0)	Retrograde
	ACT [74]	2023	309	Technical success	AUC = 0.826	Age	Connections between collateral channels and recipient vessels, channel tortuosity	Retrograde

*AUC* Area under the curve, *CCTA* Coronary computed tomography angiography, *CT-RECTOR* Computed Tomography Registry of Chronic Total Occlusion Revascularization, *ICA* Invasive coronary angiography, *CTO* Chronic total occlusion, *CTAP* CCTA plaque, *CL* Clinical and lesion-related, *CABG* coronary artery bypass graft, *CASTLE* Coronary artery bypass grafting history, ≥ 70 years of age, stump anatomy (blunt or invisible), tortuosity degree (severe or unseen), length of occlusion (≥ 20 mm), and extent of calcification (severe), *J-CTO* Japanese-chronic total occlusion, *KCCT* Korean Multicenter CTO CT Registry, *LAD* Left anterior descending, *ORA* Ostial location, Rentrop grade < 2, age ≥ 75 years, *PROGRESS-CTO* Prospective Global Registry for the Study of Chronic Total Occlusion Intervention, *RECHARGE* Registry of Crossboss and Hybrid procedures in France, the Netherlands, Belgium, and the UK, *CC* Collateral channel, *ACT* Age, connections between collateral channels and recipient vessels, and channel tortuosity

##### The CT-RECTOR score

It was the first CCTA-based score derived from 240 CTO lesions in 4 medical centers, which included 4 CT parameters (multiple occlusions, blunt stump, bending, and severe calcification) and 2 clinical variables (second attempt, duration of CTO ≥ 12 months or unknown) [42]. Each variable was assigned 1 point, and a total score was calculated by summing all points accrued. The CT-RECTOR score had excellent diagnostic performance compared to the J-CTO score in predicting guidewire passage ≤ 30 min (AUC = 0.83 vs. 0.71, *p* < 0.001). Recently, a Chinese study also verified the excellent performance of the CT-RECTOR score in predicting guidewire crossing [76].

##### The KCCT score

It was created from 4 centers of 684 CTO lesions, including 5 CT parameters (proximal blunt stump, proximal side branch, bending, occlusion length ≥ 15 mm, severe calcification or central calcification) and 2 clinical variables (second attempt, duration of CTO ≥ 12 months or unknown) [44]. Of note, calcification is subdivided into peripheral calcification (maximal encircling ≥ 180° and cross-sectional area ≥ 50%) and central calcification (cross-sectional area = 100%). Each variable mentioned above was assigned 1 point except for central calcification assigned 2 points, highlighting the important role of calcification in predicting guidewire crossing. The KCCT score showed better predictive performance than other scores (*c*-statistics = 0.78 vs. 0.65–0.72, *p* < 0.001, for all).

##### Other CT-derived scores

Recent studies [43, 77, 78] developed other CT-derived scores originally created on ICA to compare the accuracy with corresponding ICA-based scores. In the study of Fujino et al., CT-derived J-CTO showed more excellent performance in predicting successful PCI (AUC = 0.855 vs. 0.698) and guidewire crossing ≤ 30 min (AUC = 0.812 vs. 0.692) than ICA-based J-CTO [43].

#### New tools for predicting CTO PCI

With the rapid development of artificial intelligence, some new post-processing software, such as CTO quantification analysis, peri-coronary adipose tissue analysis, and radiomics, could provide more comprehensive quantitative information to help resolve this issue.

##### CTO quantification analysis

Previous studies [79, 80] with CCTA quantification software found that the high-attenuation component of CTO lesions was associated with CTO PCI outcomes. However, due to the limitation of sample size, these studies were unable to construct a predictive model that could meet the clinical application. Recently, Wang et al. [52] created the CCTA plaque score that combined the high-density plaque volume (fibrous volume + calcified volume ≥ 19.9 mm^3^) with conventional morphological and clinical parameters, yielding higher performance than other traditional scores (AUC = 0.809 vs. 0.732–0.765) due to the inclusion of objective quantitative of high-density plaques.

##### Peri-coronary adipose tissue analysis

Peri-coronary fat attenuation as a new imaging marker correlated well with coronary inflammation [81]. The peri-coronary fat attenuation around CTO lesions reflects the stability of the plaque, which can be used to predict PCI outcomes. Xi et al. [82] measured the perivascular fat attenuation index (FAI) of CTO lesions and found that the FAI in the failed antegrade PCI group was much lower than that in the successful PCI group. The cut-off value was - 77.50 HU, which combined with three morphologic parameters showed better performance than the three parameters alone (0.93 versus 0.77, *p* < 0.001). However, the study did not exclude the effect of other coronary stenosis, and further prospective study is needed in the future.

##### Radiomics

Radiomics has shown promising results in aspect of the diagnosis of vulnerable plaques and high-risk plaques [83, 84], which has been used to predict CTO PCI procedural success. Ling et al. [75] developed and validated a CT radiomics model and showed that the new model was more accurate than the CT-derived J-CTO score in predicting CTO PCI success (AUC = 0.924 vs 0.714). These findings suggest that radiomics has the potential to become a novel tool for quantifying the complexity of CTO lesions, facilitating more accurate prediction of CTO PCI outcomes.

### CCTA for preprocedural prediction of CTO PCI long-term outcomes

CCTA has the potential to predict long-term outcomes to further help risk stratification and guide clinical decision-making in CTO patients. Previous studies have shown ICA-based CTO morphological predictors or scoring systems (i.e., J-CTO score; PROGRESS-CTO score; CASTLE score; CL score) [85–88] were associated with the long-term outcomes of CTO patients after CTO PCI. Recent studies found that the series of SYNTAX scores can predict long-term outcomes in CTO patients [89, 90]. One study demonstrated that the predictive efficacy of SYNTAX score II 2020 by CCTA assessment to predict 5-year all-cause mortality was comparable to that by ICA assessment [90]. However, whether SYNTAX score II 2020 or other CTO morphological scores based on CCTA can predict long-term outcomes in CTO patients still requires further validation in larger CTO samples.

In summary, the value of preprocedural CCTA-based CTO morphology in predicting procedural success has been well-established, and the relationship between CTO morphology on CCTA and the long-term outcome has been relatively poorly studied. Therefore, a long-term prognostic prediction model based on CCTA is warranted in the future.

### CCTA for planning CTO PCI crossing strategies

The crossing strategies of CTO PCI can be generally classified into antegrade, retrograde, and hybrid approaches. The choice of crossing strategies or equipment often depends on the anatomical features of CTO lesions [56]. For example, when CTO lesions have mild calcification and tapered stump, the antegrade approach is routinely used. When the CTO lesions have length ≥ 20 mm, severe calcification, side branch, blunted stump, poor quality of the distal vessel, or proximal cap ambiguity on the CCTA that cannot be resolved, it is routinely suggested for retrograde approach, intravascular ultrasound guidance, subintimal crossing techniques, or other upgrade techniques. When collateral channels are not obvious or have severe tortuosity, retrograde approaches are not adopted routinely. Therefore, when CTO lesions are diagnosed on CCTA, radiologists can use their professional knowledge to quantify and qualify CTO lesions, thus providing interventional cardiologists with detailed anatomical features of CTO lesions and helping them choose appropriate CTO PCI strategies.

### CCTA for intraprocedural CTO PCI

In recent years, the real-time fusion of CCTA images with fluoroscopic angiography has been increasingly used to guide complex PCI, which can automatically identify coronary arteries and extract centerline using colormaps [91]. For CTO lesions, the fusion is mainly applied in highly complicated CTO lesions to resolve proximal cap ambiguity and facilitate guidewire advancement, thus reducing the need for retrograde crossing strategies [92]. The feasibility of such fusion in CTO PCI has been confirmed in previous studies [91–94]. Furthermore, implementing CCTA in the catheterization room is another emerging way to improve diagnostic and therapeutic intervention by providing an online real-time integration of CCTA data and spatial orientation [95], which has been applied in CTO PCI [96, 97]. Kim et al. [97] built a system allowing CCTA and ICA to be performed without removing patients from the table, aiming to assess the location and path of the guidewires during CTO PCI without intravascular imaging tools. They revealed that such a way had a numerically higher success rate than those without CCTA guidance (83% vs. 63%). This study showed that one-stop access to CCTA images in the catheterization room was feasible and safe in CTO patients, but this method was expensive, and the location of the guidewire cannot be identified in each case. Future studies are warranted to further confirm the feasibility and clinical benefit of the above technique and establish the appropriate protocols.

### CCTA for post-procedural CTO PCI

After successful CTO PCI, CCTA is often used to confirm successful stent placement and to follow up stent patency. Despite advances in equipment and techniques, the success rate of CTO PCI is still much lower than non-CTO PCI [5]. For those patients with previously failed CTO PCI, CCTA shows great advantages in providing detailed anatomical features for a second attempt to choose a new crossing pathway and improve the success rate of re-entry.

### Future directions

Although much progress has been made in this field, many issues need to be resolved. Firstly, CT reconstruction of CTO lesions is time-consuming and complex; a recently developed deep-learning model for automated CTO reconstruction helps solve this question [98]. However, manual measurement is still needed in this study. CTO segmentation and reconstruction are the first step; automated CTO measurement and assessment will be the next direction. Secondly, the differential diagnosis of CTO and STO is still challenging, and emerging diagnostic methods are needed to improve the diagnostic accuracy of CTO lesions on CCTA. Thirdly, clear recommendations for imaging tools and protocols for the functional evaluation of CTO patients are needed and a one-stop CT workflow for the anatomical and functional evaluation of CTO PCI still needs further validation. Fourth, with the rapid development of artificial intelligence (AI), a more accurate and automated tool based on CCTA is needed to assess the complexity of CTOs and predict CTO PCI procedural outcomes. The one-stop-shop AI platform for CTO reconstruction, diagnosis, measurement, and prediction will be a hot study direction.

## Conclusion

Diagnosis and treatment of CTO lesions are great challenges for both interventional cardiologists and radiologists. Interventional cardiologists need to consider multiple factors for CTO PCI, including clinical risk factors, evidence of ischemia, CTO anatomical parameters, and the experience of interventionalists. CCTA shows great potential for helping radiologists diagnose and evaluate CTO lesions due to its powerful post-processing technology. In addition, dynamic CTP together with CCTA that can obtain both anatomical and perfusion data can also be applied in CTO patients to help select candidates for CTO PCI. Before CTO PCI, radiologists can cooperate with interventionalists by assessing the detailed anatomical features of CTO lesions to objectively grade the difficulties of CTO PCI and then help them select the most appropriate crossing pathways. During the procedure, the feasibility of fusion of CCTA with fluoroscopic angiography technology is increasingly being demonstrated. After CTO PCI, CCTA can guide a second attempt after a failed CTO PCI or be a non-invasive follow-up tool. Taken together, CCTA is becoming a one-stop-shop imaging modality for patients with CTO.

## Data Availability

Not applicable (this is a review, not original research).

## Abbreviations

AI: Artificial intelligence
AUC: Area under the curve
CCTA: Coronary computed tomography angiography
CT-FFR: CT-fractional flow reserve
CT-RECTOR: Computed Tomography Registry of Chronic Total Occlusion Revascularization
CTO: Chronic total occlusion
CTP: CT perfusion
FAI: Fat attenuation index
ICA: Invasive coronary angiography
J-CTO: Japanese-chronic total occlusion
KCCT: Korean Multicenter CTO CT Registry
PCI: Percutaneous coronary intervention
RAG: Reverse attenuation gradient
STO: Subtotal occlusion
TAG: Transluminal attenuation gradient
TIMI: Thrombolysis in myocardial infarction
